# Geopolymers and Alkali-Activated Materials: Preparation and Properties

**DOI:** 10.3390/ma19071371

**Published:** 2026-03-30

**Authors:** Antonio D’Angelo, Michelina Catauro

**Affiliations:** Department of Engineering, University of Campania “Luigi Vanvitelli”, Via Roma 29, 81031 Aversa, Italy

The construction sector faces continuous pressure to reduce its environmental footprint while maintaining the mechanical reliability, durability, and economic feasibility required for large-scale infrastructure. Ordinary Portland cement (OPC), although indispensable in modern construction, remains a major contributor to global CO_2_ emissions and raw material depletion [1,2,3,4]. In this context, alkali-activated materials (AAMs) and geopolymers (GPs) have gained increasing attention as low-carbon alternatives [5,6,7] capable of incorporating industrial by-products (red mud, ground granulated furnace slag, fly ash, rice husk, banana peel, spent coffee, mineral wools) and hazardous waste into high-performance cementitious systems [8,9,10,11,12,13,14,15,16]. Moreover, AAMs and GPs are gaining great attention because of their versatility and applications in several fields (i.e., waste management, water treatment, catalysis, fire and heat resistance, soil stabilization, 3D printing, restoration, etc.) [17,18,19,20,21].

This Special Issue gathers eleven original research articles that collectively explore innovative processing technologies, optimized mix designs, waste valorization strategies, durability enhancement, and advanced characterization methods for sustainable geopolymer and alkali-activated systems. The contributions highlight not only incremental improvements but also transformative approaches aimed at redefining the lifecycle and functionality of construction materials.

The work of Mischinenko et al. investigates the effect of waste concrete powder content and microwave heating parameters on the properties of porous AAMs from coal gangue. In their study, the fabrication of coal gangue-based alkali-activated foams through microwave irradiation demonstrated that optimized parameters (800 W, 10 min) significantly enhanced structural stability, reduced density, and lowered thermal conductivity. The incorporation of waste concrete powder further improved overall performance while maintaining mechanical integrity, offering a route toward chemical-free foaming and reduced energy consumption [22].

Tan et al. investigated the synergistic use of industrial by-products to enhance mechanical performance and durability. The combination of steel slag powder, ground granulated blast furnace slag, and flue gas desulfurization gypsum as auxiliary materials in OPC-based systems improved compressive strength and reduced drying shrinkage in recycled cement-stabilized macadam. Microstructural investigations revealed the intergrowth of calcium silicate hydrate gel and ettringite crystals as the primary mechanism behind strength enhancement [23]. Similarly, magnesium oxide-activated slag systems reinforced with basalt fibers demonstrated higher compressive strength and freeze–thaw resistance compared to OPC-treated soft clay, with hydration products such as C–S–H gel and hydrotalcite contributing to structural integrity [24].

In one study, the role of mineral admixtures in tailoring geopolymer systems was extensively investigated. Fly ash, silica fume, and metakaolin were evaluated as partial slag replacements in sediment-based geopolymer eco-cementitious materials. While fly ash improved workability and tensile performance, silica fume significantly enhanced compressive strength, and metakaolin provided intermediate improvements. Microstructural analyses confirmed pore refinement and matrix densification through the integration of newly formed N–A–S–H and C–S–H gels [25]. The research of Xu et al. further demonstrated that the reinforcement effect of silica fume depends not only on chemical composition but also on crystallinity and intrinsic reactivity, underlining the importance of detailed precursor characterization [26].

Material behavior under complex loading conditions was investigated by Zhang et al.; in their study, modified phosphogypsum with melamine water-reducing agent was systematically studied under varying loading rates using acoustic emission monitoring, revealing rate-dependent strength enhancement and identifying acoustic precursors to macroscopic failure. This methodological integration can offer valuable insights for assessing structural reliability in dynamic environments [27].

Carollo et al. investigated viable precursors of unconventional waste streams. In particular, contaminated pharmaceutical boro-alumino-silicate glass was successfully converted into alkali-activated materials via weak alkaline attack and microwave-assisted treatment, producing mechanically stable and chemically resistant products. Firing treatments enabled either foam formation or dense glass-like materials, broadening potential applications [28].

The stabilization of galvanic sludge containing chromium and nickel within metakaolin-based geopolymers demonstrated high immobilization efficiencies, particularly when activation protocols were carefully optimized. Leaching tests confirmed the effectiveness of geopolymerization in reducing heavy metal mobility, highlighting its environmental remediation potential [29].

The incorporation of industrial dusts, red mud, and extraction sludge as 20 wt.% metakaolin substitutes showed a strong decrease in compressive strength with respect to the control without wastes. However, these waste-based geopolymers exhibited acceptable stability and promising antimicrobial properties, suggesting applications in coatings or non-structural elements [30]. Despite this, the incorporation of the same wastes as fillers in metakaolin-based geopolymers led to a strong increase in mechanical properties, suggesting that synthesis parameters strongly affect the macroscopic properties of geopolymers [31]. A fine control of synthesis parameters, as well as the AAM or GP formulation, leads to well-defined, consolidated materials with desired properties [32,33,34,35]. Furthermore, systematic pull-out testing of polypropylene, steel, and basalt fibers revealed the critical role of matrix composition in governing fiber adhesion and composite performance, with steel fibers demonstrating superior bond strength and homogeneous interfacial transition zones [36,37].

Collectively, the research presented in this Special Issue demonstrates that sustainable materials can be engineered through an integrated approach combining waste valorization, optimized activation chemistry, advanced curing technologies, and rigorous microstructural characterization. The findings confirm that performance enhancement and environmental responsibility are not mutually exclusive objectives, but rather complementary outcomes of scientifically guided material design.

The Guest Editors would like to express sincere appreciation to all authors for their valuable contributions and to the reviewers for their careful and constructive evaluations, which ensured the scientific quality of this Issue. It is our hope that the advancements reported herein will stimulate further interdisciplinary research and accelerate the translation of geopolymer and alkali-activated technologies from laboratory investigation to large-scale, standardized applications in sustainable construction.

## Abbreviations

The following abbreviations are used in this manuscript:

AAMs	Alkali-Activated Materials
GPs	Geopolymers
OPC	Ordinary Portland Cement
